# Appendicoscopy for the observation of acute appendiceal hemorrhage

**DOI:** 10.1055/a-2946-2198

**Published:** 2026-09-10

**Authors:** Zhi Peng, Faliang Xiang, Yang Yu, Xia Peng, Xuefeng Li, Rengyun Xiang

**Affiliations:** 1 Department of Gastroenterology 74680 First Affiliated Hospital of Jishou UniversityJishouHunanChina

**Fig. 1:**
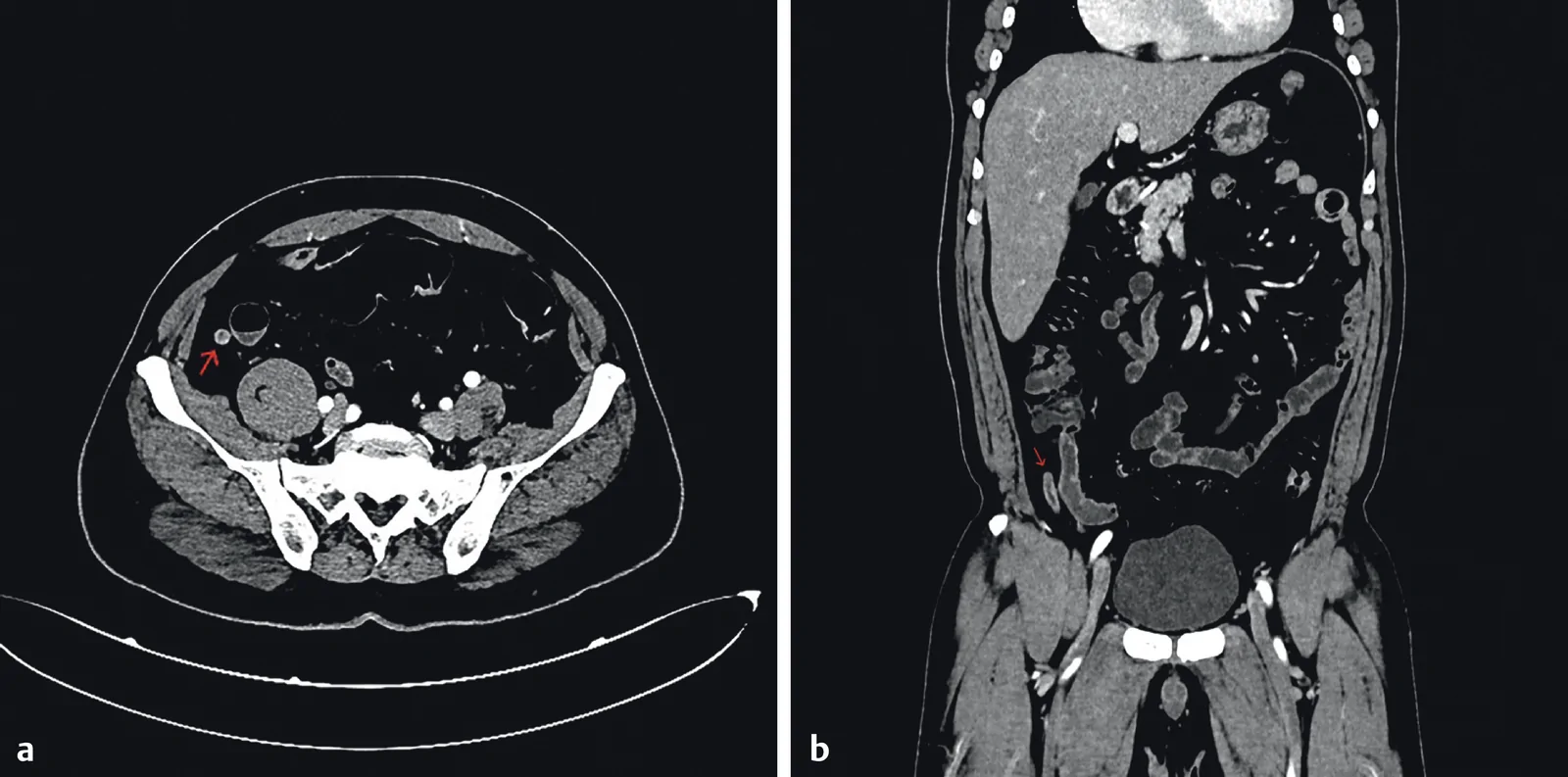
Contrast-enhanced abdominal CT scan. ( **a** ) An axial view reveals a thickened appendix. ( **b** ) Sagittal reconstruction shows an enlarged appendix.

**Fig. 2:**
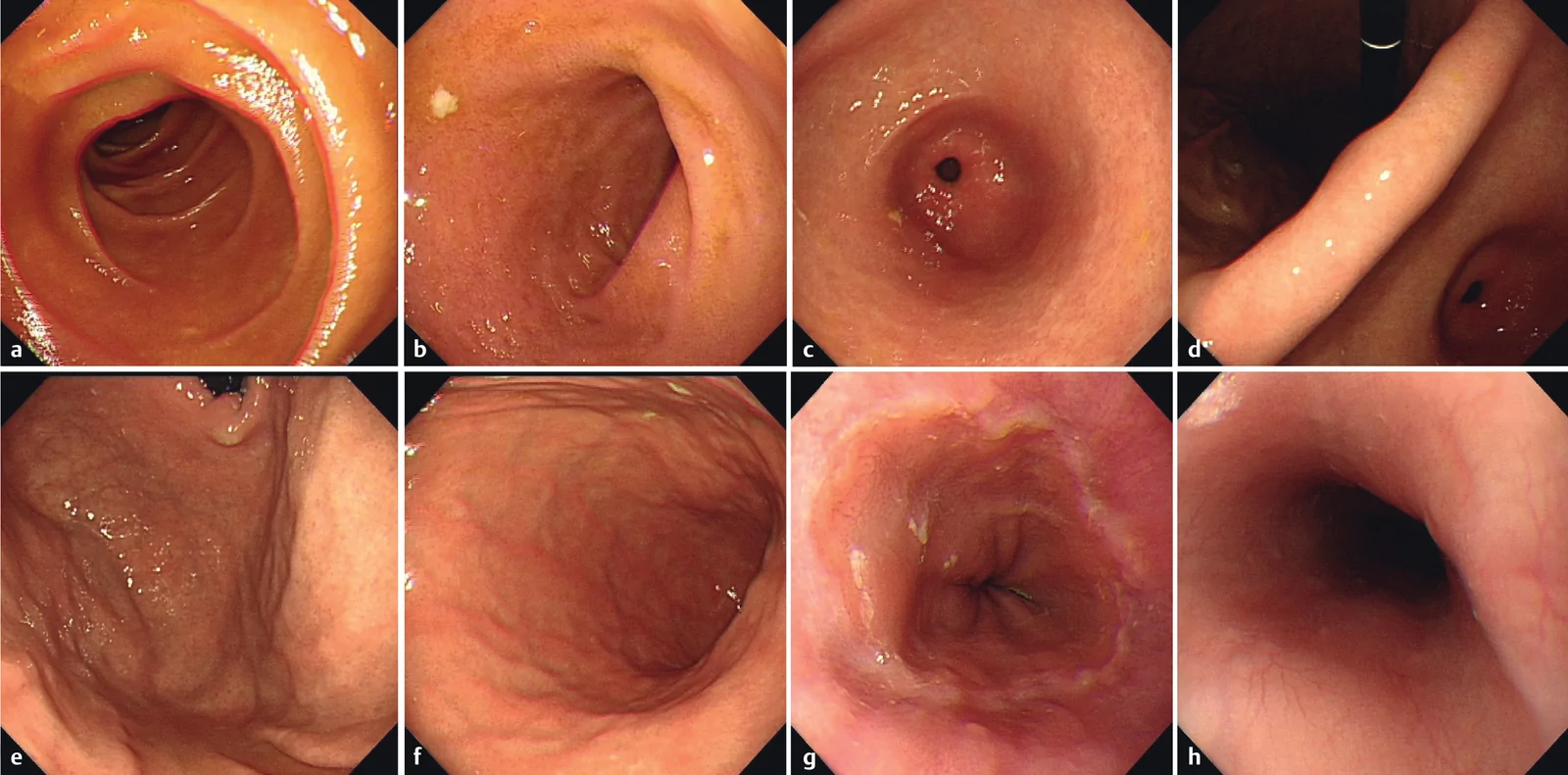
Gastroscopy showed no obvious bleeding lesions: ( **a** ) pars descendens duodeni, ( **b** ) duodenal bulb, ( **c** ) antrum, ( **d** ) gastric angle, ( **e** ) fundus of stomach, ( **f** ) gastric body, ( **g** ) cardia, and ( **h** ) esophagus.

**Fig. 3:**
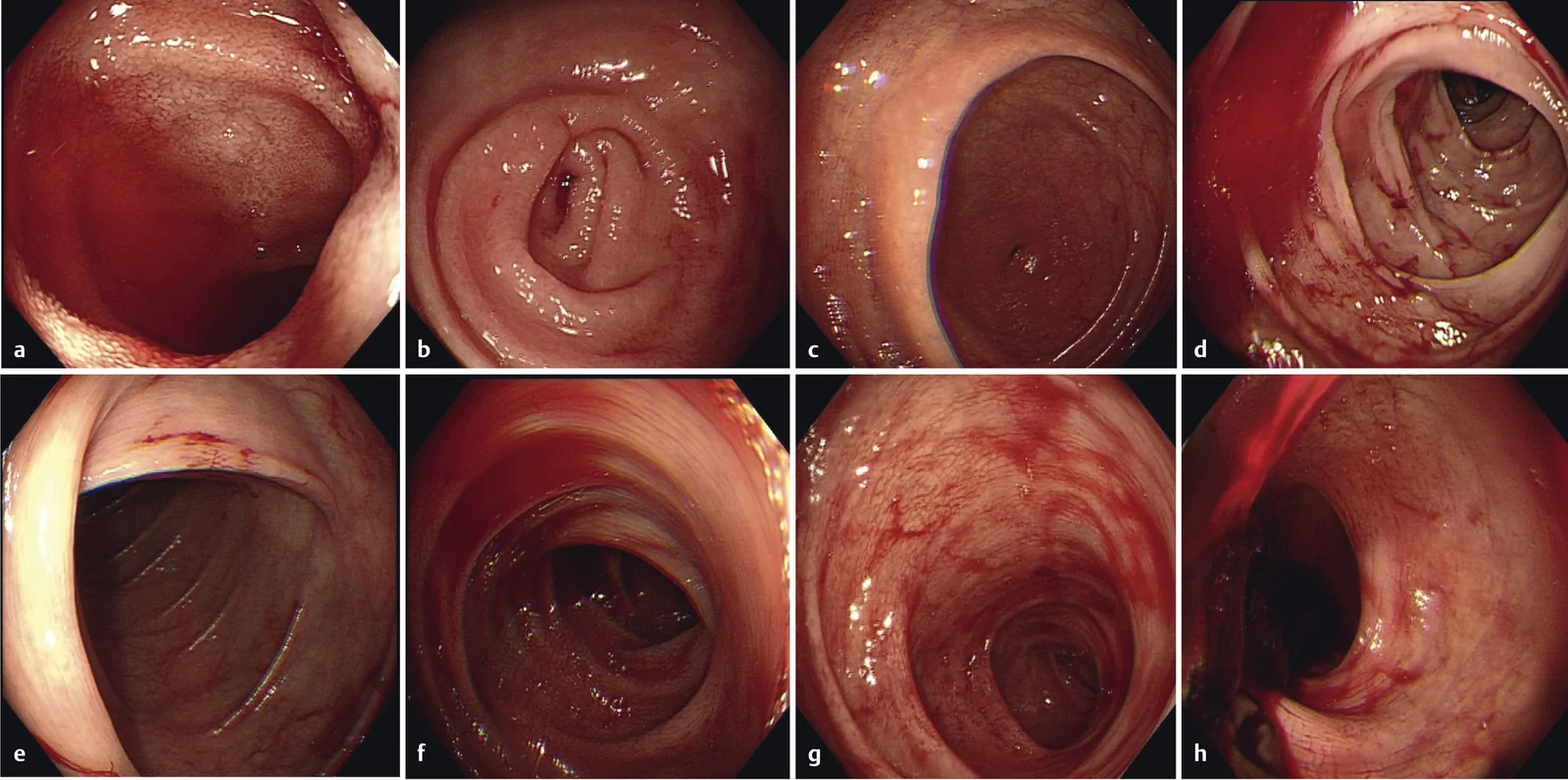
Colonoscopy revealed that there was dark red blood and a few blood clots in the colon lumen. No abnormalities were observed in the terminal ileum. ( **a** ) Terminal ileum. ( **b** ) Appendiceal orifice. ( **c** ) Cecum. ( **d** ) Colon ascendens. ( **e** ) Colon transversum. ( **f** ) colon descendens. ( **g** ) colon sigmoideum. ( **h** ) rectum.

**Fig. 4:**
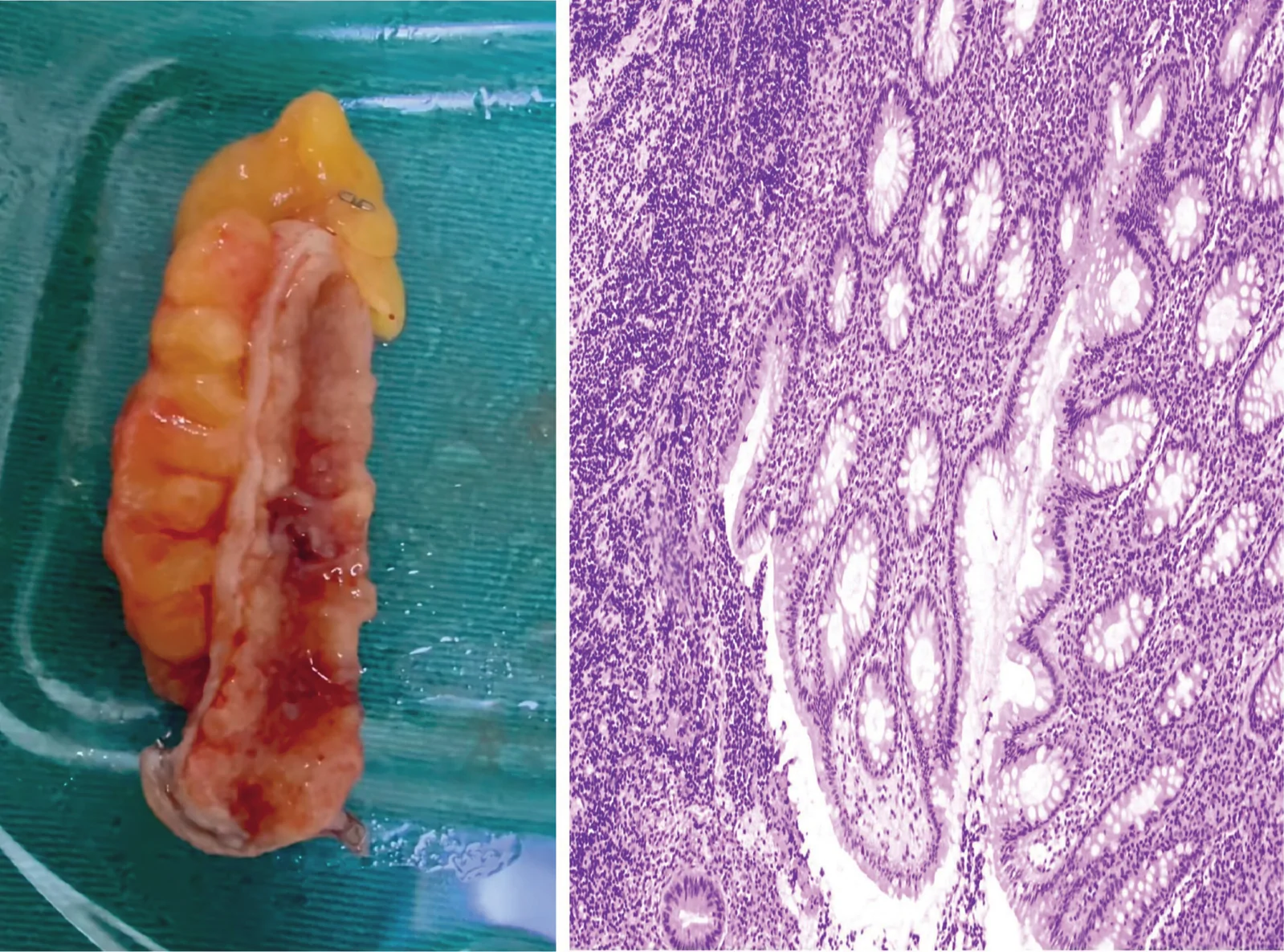
Laparoscopic and postoperative images of the appendix. ( **a** ) An appendix cut open showing an ulcer. ( **b** ) Pathological examination showed the inflammatory polyp of the appendix with ulcer formation.

A 53-year-old male patient was admitted due to hematochezia for 1 day. No abdominal pain or fever is observed. The hemoglobin level was 109 g/L on the day of admission. Abdominal computed tomography reveals that the appendix wall is focally slightly thickened, with a thickness of approximately 3.4 mm, and enhancement is observed on contrast-enhanced scanning ( Fig. 1 ). No obvious bleeding lesion was observed via gastroscopy ( Fig. 2 ). Colonoscopy revealed dark red blood fluid and a small amount of blood clots in part of the colon lumen, No abnormalities were observed in the terminal ileum ( Fig. 3 ). Upon careful examination, fresh blood was seen flowing out from the appendiceal orifice, confirming appendiceal bleeding. The appendoscopy is inserted through the biopsy channel, and enters the appendiceal lumen through the appendiceal orifice with an insertion depth of approximately 8 cm. Hematocele is observed in the appendiceal lumen. After repeated water injection for irrigation and observation, a neoplasm is identified in the appendiceal lumen, with surface ulceration and blood clot attachment ( Video 1 ). Laparoscopic appendectomy was performed emergently. Postoperative pathological examination showed the inflammatory polyp of the appendix with ulcer formation ( Fig. 4 ). No recurrent hematochezia was observed during the postoperative follow-up.

**Video 1** Colonoscopy revealed fresh blood flowing from the appendiceal orifice. Upon entering the appendiceal lumen via appendoscopy, a neoplasm was identified, with ulceration and blood clots attached to its surface.


Reports of lower gastrointestinal bleeding originating from the appendix are extremely rare, and the etiology of such bleeding remains incompletely understood. Appendectomy is the first-line approach for both treatment and definitive etiological diagnosis. 1​ Given the narrow lumen of the appendix, conventional endoscopy can hardly enter the appendiceal lumen to visually identify the exact etiology of hemorrhage. There have been reports on the insertion of ultra-thin gastroscopes into the appendiceal lumen for observation. However, inserting an ultra-thin gastroscope into the appendiceal lumen requires the operator to possess extremely high technical proficiency, which makes it difficult to rapidly identify the cause of bleeding in emergency situations. 2​ In this case, we utilized appendoscopy to rapidly access the appendiceal lumen, directly identify and confirm the bleeding lesion, thereby improving the diagnostic accuracy for appendiceal lesions.

Endoscopy_UCTN_Code_CCL_1AD_2AF
